# “I’M HAVING THE WORST TIME”: CAREGIVER EXPERIENCES WHEN OLDER ADULTS UNDERGO MAJOR ELECTIVE SURGERY

**DOI:** 10.1093/geroni/igae098.1761

**Published:** 2024-12-31

**Authors:** Ellis Dillon, Christina Keny, Lingsheng Li, Veronica Yank, Alexis Colley, Malini Ramaiyer, Victoria Tang

**Affiliations:** University of Connecticut, Farmington, Connecticut, United States; University of California San Francisco, San Francisco, California, United States; University of California San Francisco, San Francisco, California, United States; University of California San Francisco, San Francisco, California, United States; University of California San Francisco, San Francisco, California, United States; Johns Hopkins University, Baltimore, Maryland, United States; University of California San Francisco, San Francisco, California, United States

## Abstract

Older adults are at higher risk of major complications, functional decline, and mortality after surgery. Family caregivers play a critical role supporting older adults, but often experience caregiver burden and distress. We know little about family caregiver experiences before and after a major operation. We conducted semi-structured interviews at three timepoints (2-weeks before and 1 and 3-months post-operation) with family caregivers of adults ≥ 65 years undergoing a major elective operation at an academic hospital. Data were analyzed using inductive thematic analysis. Thirteen family caregivers completed 27 unique interviews. Family caregivers mean age was 67 (range 44-79), 67% identified as White, and 61% were female spouses. Cancer was the most common indication for operations. Themes identified included: (1) the caregiving role evolves to become all consuming - “my day is kind of wrapped around what he needs,” (2) caregiving demands may disrupt the relationship dynamic - “you just become more of a caregiver than a wife,” (3) uncertainties about surgery and caregiving demands contribute to distress - “I’d like to know the statistics and if there was a 25% chance for full recovery,” and (4) family caregiver insights about how to support their own well-being through better guidance and support through all phases of surgical care. Family caregivers for older adults undergoing a major operation face many challenges and need better psychosocial and instrumental support, family-centered education, and communication. By identifying and addressing these needs, we can optimize the well-being of family caregivers and the older adults they support.

